# Semi-Automatic Classification of Skeletal Morphology in Genetically Altered Mice Using Flat-Panel Volume Computed Tomography

**DOI:** 10.1371/journal.pgen.0030118

**Published:** 2007-07-20

**Authors:** Christian Dullin, Jeannine Missbach-Guentner, Wolfgang F Vogel, Eckhardt Grabbe, Frauke Alves

**Affiliations:** 1 Department of Diagnostic Radiology, Georg-August-University, Göttingen, Germany; 2 Department of Hematology and Oncology, Georg-August-University, Göttingen, Germany; 3 Department of Laboratory Medicine and Pathobiology, University of Toronto, Toronto, Ontario, Canada; Maine Center for Osteoporosis Research, United States of America

## Abstract

Rapid progress in exploring the human and mouse genome has resulted in the generation of a multitude of mouse models to study gene functions in their biological context. However, effective screening methods that allow rapid noninvasive phenotyping of transgenic and knockout mice are still lacking. To identify murine models with bone alterations in vivo, we used flat-panel volume computed tomography (fpVCT) for high-resolution 3-D imaging and developed an algorithm with a computational intelligence system. First, we tested the accuracy and reliability of this approach by imaging discoidin domain receptor 2- (DDR2-) deficient mice, which display distinct skull abnormalities as shown by comparative landmark-based analysis. High-contrast fpVCT data of the skull with 200 μm isotropic resolution and 8-s scan time allowed segmentation and computation of significant shape features as well as visualization of morphological differences. The application of a trained artificial neuronal network to these datasets permitted a semi-automatic and highly accurate phenotype classification of DDR2-deficient compared to C57BL/6 wild-type mice. Even heterozygous DDR2 mice with only subtle phenotypic alterations were correctly determined by fpVCT imaging and identified as a new class. In addition, we successfully applied the algorithm to classify knockout mice lacking the DDR1 gene with no apparent skull deformities. Thus, this new method seems to be a potential tool to identify novel mouse phenotypes with skull changes from transgenic and knockout mice on the basis of random mutagenesis as well as from genetic models. However for this purpose, new neuronal networks have to be created and trained. In summary, the combination of fpVCT images with artificial neuronal networks provides a reliable, novel method for rapid, cost-effective, and noninvasive primary screening tool to detect skeletal phenotypes in mice.

## Author Summary

Transgenic mice are key models to shed new light on gene function during development and disease. Reliable high-throughput screening tools will facilitate the identification of transgenic mice with distinct phenotypes. In particular, alterations of the skull are difficult to detect by visual inspection due to its very complex morphological structure. Here, we used high-resolution flat-panel volume computed tomography (fpVCT), a novel semi-automatic screening tool to image skull-shape features of mice. The resulting 3-D datasets were combined with artificial neuronal networks and complex nonlinear computational models to permit rapid and automatic interpretation of the images. Compared to the extremely laborious landmark-based analysis, the manual work in our approach was reduced to the control of skull segmentation of images obtained by fpVCT. We applied our approach to genetically altered mice and various mouse strains and showed that it is an accurate and reliable method to successfully identify mice with skeletal phenotypes. We suggest the new methodology will also be a valuable tool for an in vivo, rapid, cost-effective, and reliable primary screen to identify skull abnormalities generated by random mouse mutagenesis experiments.

## Introduction

Following the sequencing of the mouse and human genomes, attention has now focused on assessing gene function by gain-of-function mutations or targeted deletion of genes to address their function in vivo. However, many transgenic or knockout mice display a mild pathology without overt phenotypic alterations, which is clearly of utmost importance in understanding human diseases. This, in turn, has created an enormous demand for effective tools to assess the phenotype of mouse models so that gene expressions can be understood in a biological context [1]. However, the development of high-throughput mouse mutagenesis protocols requires a time- and cost-effective mode for primary testing of phenotypes.

In previous work, noninvasive imaging techniques such as computed tomography (CT) and magnetic resonance imaging have been applied to the anatomical phenotyping of transgenic mouse embryos [2–4] as well as in the brain and skulls of mouse models [5–7]. The measurement of 3-D coordinates as biological landmarks on the skull was used to analyze craniofacial phenotypes in mouse models for Down syndrome [8]. Similarly, metabolic profiling of cardiac tissue through high-resolution nuclear magnetic resonance spectroscopy in conjunction with multivariate statistics was used to classify mouse models of cardiac disease [9].

These imaging technologies for rapid visualization of large regions of anatomical structures have several important advantages over classical histology. The differential comparison of a large dataset of images using traditional radiological observation and a well-trained eye, especially between complex skeletal structures, is often inadequate. Therefore, automated analysis of images to detect skeletal phenotypes in mouse models will be highly advantageous.

Here, we have performed flat panel-based volume computed tomography (fpVCT) for rapid high-resolution imaging of bone structures in combination with artificial neuronal networks (ANNs) that are complex nonlinear computational models, designed much like the neuronal organization of a brain [10–15]. These networks are composed of a large number of highly interconnected processing elements, termed neurons, working in parallel order to model complicated biological relationships without making assumptions based on conventional statistical distributions. Neuronal networks learn by example so the details of how to recognize the phenotype of the skull are not needed. What is needed is a set of examples that are representative of all the variations of the phenotype [12,13]. Such neuronal networks have already been applied to characterize the variability of anthropological features of the human nasal skeleton [14] and to analyze and classify human craniofacial growth [15]. Here, fpVCT imaging enables the 3-D visualization of small anatomic details of bone structures. By selecting subvisual information from these fpVCT datasets of the skull, we applied ANNs to predict skeletal phenotypes in mouse models.

For visualization of the feature space structure, here, we analyzed the automatically generated skull-shape features with principle component analysis (PCA) and cluster analysis. PCA simplifies multidimensional datasets to lower dimensions and consequently transforms them into orthogonal linear to a new coordinate system such that the greatest variance comes to lie on the first coordinate, the second greatest variance on the next coordinate, and so on [16]. Cluster analysis is the partitioning of data into subsets, so that the data in each subset share some common traits with the use of some defined distance measurements [16,17].

The method was applied to knockout mice of a subfamily of tyrosine kinase receptors, discoidin domain receptors (DDRs), which are selectively expressed in a number of different cell types and organs; upon collagen activation DDRs regulate cellular adhesion and proliferation as well as extracellular matrix remodeling [18,19]. Lack of DDR2 resulted in reduced chondrocyte proliferation and shortening of long bones and the snout [20]. In contrast little is known about skeletal abnormalities of DDR1-deficient mice [21].

The purpose of this study is to present a rapid method for primary screening of skeletal phenotypes using fpVCT, allowing detailed nondestructive imaging of the skull in vivo. Using skull-shape features semi-automatically calculated from fpVCT datasets in combination with ANNs, we were able to successfully classify adult knockout mice with various bone malformations as well as identify mouse populations with subtle skeletal abnormalities with high accuracy.

## Materials and Methods

### Animals

All animals were maintained under pathogen-free conditions and housed in accordance with German animal welfare regulations. All animal protocols were approved by the administration of Lower Saxony, Germany. For this study homozygous and heterozygous DDR1- and DDR2-deficient mice on inbred C57BL/6 backgrounds, five DDR1/2 double knockout (DDR1−/−//DDR2−/−) mice, as well as C57BL/6 wild-type and severe combined immunodeficient (SCID) mice strain CB-17/ *Ztm-scid,* of different ages and sexes were used. To allow nearly complete ossification of the skull, all mice with the exception of the 14-d-old double knockout mice were older than 50 d. For this study we used 85 mice in total: 29 DDR1/2+/+, 25 DDR2−/−, ten DDR2+/−, nine DDR1−/−, five DDR1−/−//DDR2−/−, and seven SCID mice. For descriptive statistics, see Table 1.

**Table 1:**
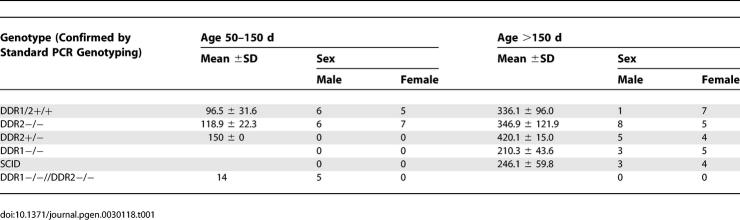
Collection of Mice

The genotype of mice was verified by standard techniques. DNA was isolated from tail biopsies, and polymerase chain reaction (PCR) was performed as described previously [20,21]. These results were compared to the outcome obtained by fpVCT datasets in combination with an ANN.

### Imaging Protocol by fpVCT

Mice were anesthetized with vaporized isoflurane at 0.8 − 1% concentration throughout the imaging session and centered on the fpVCT gantry axis of rotation. The fpVCT prototype used in this study was developed and constructed by General Electric Global Research (http://www.ge.com/research). It consists of a modified circular CT gantry and two amorphous silicon flat-panel X-ray detectors, each of 20.5 × 20.5 cm^2^ with a matrix of 1,024 × 1,024 detector elements and a resolution of 200 μm. The fpVCT uses a step-and-shoot acquisition mode. Standard z coverage of one step is 4.21 cm. The mice were placed perpendicular to the z-axis of the system in order to scan the whole mouse with one rotation. All datasets were acquired with the same protocol: 1,000 views per rotation, 8-s rotation time, 360 used detector rows, 80 kVp, and 100 mA. A modified Feldkamp algorithm was used for image reconstruction resulting in isotropic high-resolution volume datasets (256 × 256 matrix, resolution about 200 μm).

### Characterization of Skulls

**Figure 1:**
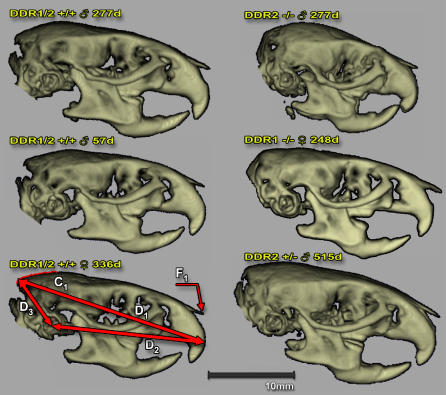
Comparison of Skull Image Presented is a volume rendering of skull forms of C57BL/6 wild-type mice of different sexes and ages (left panel) and DDR2−/−, DDR1−/−, and DDR2+/− mice (right panel). Displayed are distances and representative features of the skeleton of the head viewed from the right side. *D*_1_ represents the maximal distance between the external occipital protuberance and the incisor teeth. The distance between the center of the inner ear and the incisor teeth is labeled *D*_2_, whereas *D*_3_ delineates the distance between the center of the inner ear and the external occipital protuberance. *C*_1_ depicts the bend of parietal, interparietal, and occipital bone and is measured as sum of the local contour curvature. The landmark *F*_1_ symbolizes the nasal bone.

To characterize the skull differences between the various mouse lines, we used landmark-based geometric analysis. Distances on the side-view-projection of the 3-D rendered mouse skull (*D*_1_, *D*_2_, and *D*_3_) as well as the curvature of the occipital region (*C*_1_) were manually measured (Figure 1). To calculate the curvature, the contour part between the inflection point on the superior region of the head as well as the inflection point after the sharp bend on the occipital region were manually isolated (Figure 1). For comparability of curvature values, all contours were stretched to the same length. Contours were represented in Freeman code (chain code). Therefore, each of the eight possible directions from one to the next contour point was encoded by a number from 0 to 7. We started with 0 on the right and used increasing numbers in a clockwise orientation. Since the determination of the local curvature from the changes of these direction codes results in rather noisy curves [22], we calculated the local curvature for a surrounding of six contour points, three forward and three backward of the actual point [23]. *C*_1_ represents the sum of these curvature values.

### Segmentation and Preprocessing of Datasets

Segmentations of skulls were done using a simple threshold algorithm in the head region in order to segment all voxel with values above the threshold. For this purpose all parts of the segmentation that do not represent the skull were removed. All segmented voxel were referenced by the letter S.

Skull orientation and size were standardized by computing the centroid (Equation 1) and mass centroid axis, which are the eigenvectors of the inertial tensor (Equation 2), and rotating the skull in a such way that the x-axis points along the main mass centroid axis. To calculate the inertial tensor, the mass of a voxel is required. Bone consists of structures expressing different CT-numbers, and skull is surrounded by soft tissue that is characterized by low CT-numbers. Therefore, a distribution of these CT-numbers with a left skew gauss-like shape due to the partial volume effect was observed. The real surface of the bone is within a surface voxel. Because surface voxel must not contribute to features with the same magnitude as voxel representing solid bone, we determined the mass of a voxel by the weight function *g*(v) (Equation 3). Applying this equation voxel with a density equal to the mean density of bone had more influence in further calculations than others.

Mass centroid:

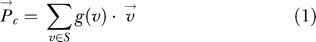



Inertial tensor:

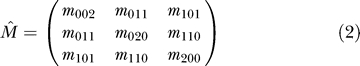



Weight function:

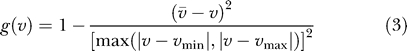



Moments:

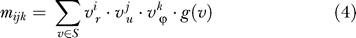



*v*_r_ = radius component, *v*_υ_ = polar angle, and *v*_φ_ = azimuth angle of position vector *v*

Maximal dimensions along every axis were determined after the segmentation procedure and skulls were isotropically rescaled between −1 and 1 in all dimensions. These transformed skulls provide a basis to calculate size- and orientation-independent form features.

### Calculation of Skull-Form Features

In a global depiction the components of the inertial tensor (Equation 3) are second-order moments. Here we used as features the moments (Equation 4) up to an order of four [24]. Since mouse skulls can be described as ellipsoid, these moments are not generated in Cartesian but in spherical coordinates. With the index of formula (Equation 4) we obtained the following 34 moments: m_001_, m_010_, m_100_, m_002_, m_020_, m_200_, m_011_, m_101_, m_110_, m_003_, m_030_, m_300_, m_012_, m_102_, m_021_, m_120_, m_201_, m_210_, m_111_, m_004_, m_040_, m_400_, m_013_, m_103_, m_031_, m_130_, m_301_, m_310_, m_022_, m_202_, m_220_, m_112_, m_121_, and m_211_.

If we consider Cartesian coordinates, it is possible to compare the second-order moments with a mass distribution away from a rotation axis. This means that a high second-order moment indicates that the mass of parts of the skull is distributed farther from the dedicated axis. Assuming that the mass density is nearly the same between the different skulls, a higher value second-order moment connotes the skull being more expanded perpendicular to that axis. Third-order moments impact the aberration from rotation symmetry perpendicular to the dedicated axis, and fourth-order moments impact the outliers. Since these predictions can be applied accordantly for spherical coordinates, form features were now encoded in these statistical parameters, in total 34 moments, independent of skull size and orientation. Sex of the animal (0 = female and 1 = male) and the normalized age, *af* (Equation 5) were added as features 35 and 36.

The feature *af* was calculated under the assumption that ossification of the skull has an exponential behavior and is up to 90% completed after 60 days [25]. So the codomain of (Equation 5) is the interval (0, 0.9] for mice that are between 1 and 60 d old.

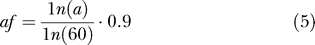

*a* = age in days.

Finally, all features were transformed in an interval [−1, 1] to raise the stability of the training process of the artificial networks. For segmentation and feature calculation purposes, the algorithms were implemented using MSVC++ 6.0 and the additional libraries QT4.2 (Trolltech, http://www.trolltech.com) and VGL2.4 (Volume Graphics GmbH, http://www.volumegraphics.com). A C++ implementation of transformation and feature generation part is presented in Protocol S1.

### Neuronal Networks: Basic Structure

To assess mouse phenotypes by skull images, several different ANNs were initially tested. Here, skull-form features were used to train multilayer perceptron network models developed with the Stuttgart Neuronal Network Simulator (SNNS) in context with a back-propagation-learning rule [26]. A multilayer perceptron consists of one input layer, some hidden layers, and an output layer. Such a network performs a transformation from an input pattern, which is the summary of all input values applied to the input neurons, into an output pattern and can be used for classification purposes by assigning classes to the output pattern. Hidden layers do not interact with the outside and are only necessary to perform the classification process.

For training and validation of a neuronal network, at least two different classified datasets have to be applied: one for the training process in order to impact the needed transformation between input pattern and the known output pattern in the network via the learning rule and one to test the network. The latter set consists of data not presented during the learning process. To test for accuracy, all network responses for the test dataset were compared manually with the known output pattern validated by genotyping of the mice. Here, the training dataset contains images from a minimum of three times more mice than the test dataset and the distribution of the pattern between these two datasets were chosen randomly to avoid bias.

### Statistical Analysis

For statistical analysis we performed PCA, cluster analysis, and Student's *t*-test. PCA and clustering analysis were done with PAST, free statistics software [27]. We used an agglomerative hierarchical cluster method with a Euclidean metric that groups step-by-step samples together with the lowest distance. The result of the algorithm is displayed as a dendrogram.

Furthermore, to determine whether the means of measured distances and curvatures between two mice strains differ significantly, we used the unpaired Student's *t*-test performed online with http://www.physics.csbsju.edu/stats/t-test.html. All data were expressed as mean ± SD, and statistical significance was established at a *p*-value less than 0.05.

## Results

### Anatomical Phenotypes of Transgenic Mice

Noninvasive imaging by fpVCT with 200 μm isotropy and 8-s scan times enabled the selective 3-D visualization of the skull of individual adult DDR1-and DDR2-deficient mice as well as wild-type littermates (Figure 1). Image comparison by visual inspection of C57BL/6 wild-type mice of different sex and age demonstrated minor interindividual differences within the complex skull morphology formed by the cranium and mandible. These minor variations in skull shapes are due to sexual dimorphisms and age dependent ossification. Individual skulls of mice from the control strain differ mainly in relative size and are characterized by the forming of the tympanic bulba, the infraorbital hiatus, and the grade of ossification of the frontal and occipital bone (Figure 1, left panel).

**Figure 2:**
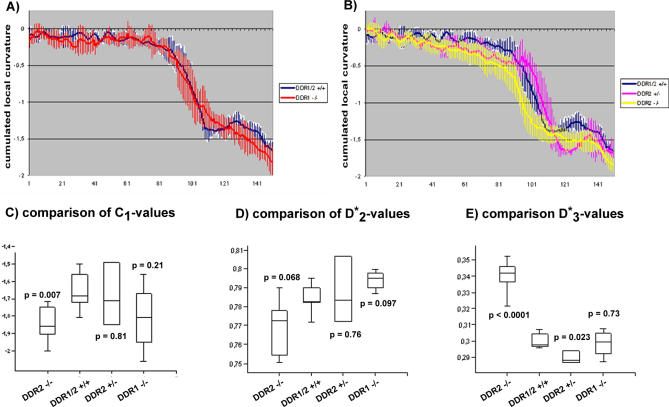
Statistical Evaluation of Various Metrical Skull Features (A, B) Progression of the cumulative local contour curvatures *C*_1_ follows the contour positions of knockout mice and their controls. Shown are the mean values and the local standard deviations for each mice strain. (C–E) Box plots for *C*_1_, *D* ^*^_2_, and *D* ^*^_3_ values are shown. *p*-Values for the paired Student's *t*-test for each feature of every strain are indicated in comparison to wild-type controls. Differences with *p* < 0.05 were considered to be significant. Note, that parameters *C*_1_ and *D* ^*^_3_ in DDR2-deficient mice, *D* ^*^_3_ in heterozygous DDR2-deficient mice, and *D* ^*^_2_ in DDR1-deficient mice differ significantly in comparison to wild-type controls.

Representative examples of skull images from the various knockout mice in comparison to controls are shown in Figure 1, right panel. For comparative analyses of the various skulls we introduced landmarks located on the cranium that show marked differences mainly between DDR2-deficient mice and wild-type controls. Curvature *C*_1_, located at the posterior neurocranium, depicts the bend of parietal, interparietal, and occipital bone. As plotted in Figure 2A and 2B the local curvature is a nonconstant function, which differs between the knockout mice on different contour positions. We introduced *C*_1_ as the sum of the local curvature values representing the value of the cumulative curves on the maximal contour position. *D*_1_ represents the maximal distance between the external occipital protuberance and the incisor teeth. The distance between the center of the inner ear and the incisor teeth is labeled with *D*_2_, whereas D_3_ is the distance between the center of the inner ear and the external occipital protuberance (Figure 1, left panel). To take into account different skull sizes, we introduced new parameters such as *D* ^*^_2_ and *D* ^*^_3_, which are calculated from *D*_2_ and *D*_3_ by dividing the data with the total skull distance of *D*_1_. The nasal bone is represented by *F*_1_.

The neurocranium of DDR2-deficient mice displays a more round shape in comparison to controls illustrated by an increase in the magnitude of curvature *C*_1_ (Figure 1, right panel). This is clearly depicted by different cumulative curvatures of *C*_1_ especially in the late part of the curve of DDR2-deficient mice in comparison to their wild-type controls (Figure 2B and 2C). Skulls of DDR2−/− mice are jolted as shown by a reduced length of *D*_1_ (20.31 ± 0.58 mm versus 22.68 ± 0.45 mm for control). Furthermore a spherical skull shape is characteristic for DDR2-deficient mice as demonstrated by a reduced mean value for *D* ^*^_2_ (0.77 ± 0.016 versus 0.784 ± 0.009 for control; *p* = 0.068; Figure 2D) and a significant increased value for *D* ^*^_3_ (0.34 ± 0.009 versus 0.30 ± 0.005 for control; *p* < 0.0001; Figure 2E). Characteristic of DDR2-deficient mice is the nasal bone demonstrated by the landmark *F*_1_ that appears to be altered and of different shape in comparison to controls by visual inspection (Figure 1, right panel).

Alterations between wild-type and heterozygous DDR2-deficient mice are very subtle and can hardly be depicted by comparative morphological analysis using traditional radiological observation (Figure 1). This is shown by no significant differences of *D* ^*^_2_ (0.79 ± 0.018 versus 0.78 ± 0.009 for control; *p* = 0.76; Figure 2D) and the curvature *C*_1_ (−94.98 ± 14.30 versus −84.96 ± 8.56 for control; p = 0.81, Figure 2C). Only the value for *D* ^*^_3_ was significantly altered in DDR2 +/− mice (0.29 ± 0.004 versus 0.30 ± 0.005 for control; *p* = 0.023; Figure 2D).

DDR1-deficient mice are known to be smaller. Here, one characteristic feature of the skull of DDR1-deficient mice is an altered curvature progression compared to their wild-type controls (Figure 2A). However, the value for *C*_1_ (−90.24 ± 4.29 versus −84.96 ± 8.56 for control; *p* = 0.21) was not significantly different (Figure 2C). Distance measurements also revealed no significant differences to the values obtained in wild-type controls. *D* ^*^_2_ was reduced in wild-type mice (0.80 ± 0.005 versus 0.78 ± 0.009 for control; *p* = 0.097) whereas *D* ^*^_3_ was within the same range (0.30 ± 0.009 versus 0.30 ± 0.005 for control; *p* = 0.73; Figure 2D and 2E)

Comparative landmark based analysis of skulls of DDR2-deficient mice confirmed statistical significant bone deformations compared to controls, whereas analysis in DDR1-deficient mice determined no skull alterations.

### Analysis of the Generated Feature Space for Different Knockout Mice with PCA and Cluster Analysis

**Figure 3:**
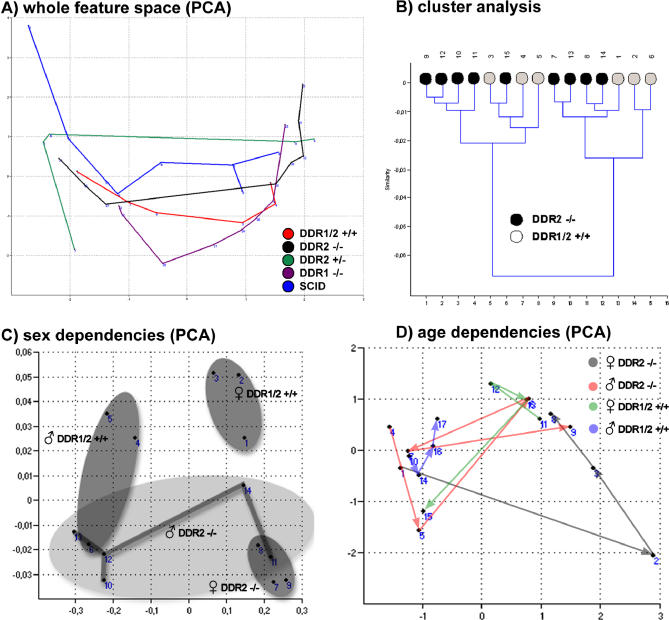
Statistical Evaluation of the Generated 34-D Skull-Shape Features (A) A scatter plot is demonstrated for the two main components of the PCA-transformed 34-D feature space of all skull-shape features. The samples for each mice strain are interconnected with lines. The clusters are strongly overlapping, and in this case the two dimensional subspaces are not linearly separable. (B) A further cluster analysis for DDR2−/− and DDR1/2+/+ mice based on the Euclidean distance between the feature vectors does not separate the two groups. (C) PCA-transformed skull-shape features of a subset of age-matched DDR1/2+/+ and DDR2−/− mice with different sex are well clustered according to their sex for DDR1/2+/+ and the female DDR2−/− mice. The male DDR2−/− mice are widely separated resulting in overlaps with their females. (D) PCA of DDR2−/− and DDR1/2+/+ mice that were separated in male and female are illustrated. Sample points of each group are connected with arrows in the direction of increasing age. No correlation between age and feature vector was depicted.

PCA and cluster analysis were performed in the complete 34-D feature space to visualize and evaluate its structure and the existence of clusters corresponding to distinct mice types. Figure 3A displays the two-dimensional subspace for the two main components of the PCA for a subset of mice. For better visualization sample points of one class were interconnected with lines. No cluster could be detected, indicating a poor conditioned feature space. Cluster analysis applied to skull features of DDR2-deficient mice, and their wild-type controls do not discriminate between the two mouse populations (Figure 3B).

To evaluate the influence of sex- and age-dependent skull-shape differences, we performed PCA for an age- and a sex-matched subset, respectively, using datasets from DDR2-deficient mice and their wild-type controls (Figure 3C and 3D). Skull shapes of both male and female controls and female DDR2−/− mice displayed strong sex related differences shown by higher interclass distances in comparison to intraclass distances. Especially, control mice were grouped into two subsets of male and female mice. Only the cluster of the DDR2-deficient mice was very widespread, thus overlapping the clusters of female DDR2−/− and male control mice. Excluding male DDR2−/−, the feature space can be divided into two half planes for male and female mice. Interestingly, the male DDR2-deficient mice can be subdivided into two subgroups consisting of male and female “looking” mice (Figure 3C). In the PCA of the age dependency of the feature vectors including young mice, we depicted age related varieties, but were not able to determine any functional relationship between increase in age and the position of the feature vector (Figure 3D).

Since the skull-shape features used in this study are dependent on sex, we applied in further experiments both female and male mice to train the ANNs. In order to suppress the age dependent effects we only used mice older than 50 days with the exception of DDR1−/−//DDR2−/− mice, which were scanned ex-vivo when 14 d old.

### ANN 1 Applied to fpVCT Datasets Allows Phenotypic Assessment of DDR2-Deficient Mice

**Figure 4:**
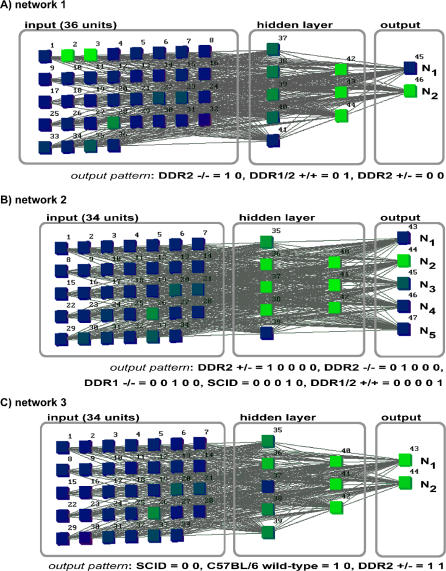
Architectures of the ANNs 1 to 3 to Discriminate DDR2−/−, DDR2+/−, DDR1−/−, DDR1/2+/+, and SCID Mice between Different Mouse Populations (A) Network 1 for paired classification classifies DDR2-deficient mice against their wild-type littermates. It consists of one input layer with 34 neurons for the skull-shape features and two additional input neurons for age and sex. The image shows the network response for a DDR1/2+/+ mouse (pattern 4, Table 2) (B) Neuronal network 2 applied for classification of all five mice strains in a mixed collective consists of one input layer with 34 neurons, two hidden layers with five and three neurons, and one output layer with five neurons *N*_1_ − *N*_5_. In network 2 the taught output identifies each output neuron as one mouse population. The output neuron with the highest activation is called the “winning” neuron and indicates with which class of mice the input pattern is associated. The network response for input pattern 4, the input of skull-shape features of a DDR2−/− mouse is displayed. This is demonstrated by high activation of the output neuron *N*_2_ (green, high activation; blue, low activation). (C) Neuronal network 3 applied for classification of DDR2+/− mice, which were incorrectly identified in network 2 as C57BL/6 control mice, consists of one input layer with 34 skull-based features, one hidden layer with five neurons, and one output layer with the neurons *N*_1_ and *N*_2_. The output of an output unit is a value between 0 and 1. The combination of the taught output that represents a distinct phenotype of DDR2+/− and SCID or C57BL/6 wild-type mice is shown below. High activation of output neuron *N*_1_ with simultaneous low activation of *N*_2_ represents C57BL/6 wild-type mice, low activation of both *N*_1_ and *N*_2_ represents a SCID mouse, and low activation of *N*_1_ by simultaneous high activation of *N*_2_ represents a DDR2+/− mouse. The network response for input pattern 4, the input of skull-shape features of a heterozygous DDR2 mouse, is displayed. This is demonstrated by high activation of *N*_1_ and *N*_2_ (green, high activation; blue, low activation).

ANN 1 shown in Figure 4A was developed to identify DDR2-deficient mice displaying a known skeletal phenotype between control littermates. Neuronal network 1 received one import layer with 36 neurons to import the skull features calculated from fpVCT datasets. It consists of two hidden layers with five and three neurons and of one output layer with two neurons, *N*_1_ and *N*_2_. All neurons were connected with short cuts and trained with the back-propagation momentum-learning rule.

**Table 2:**
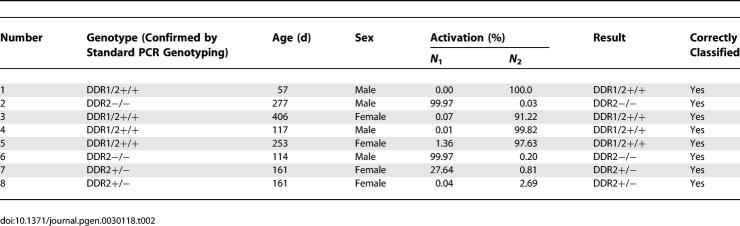
Phenotypic Assessment of DDR2-Deficient Mice Using fpVCT Datasets and ANN 1

A total of three different phenotypic assessments corresponding to DDR2−/−, DDR2+/−, and control mice were encoded in the output pattern as demonstrated in Figure 4A. We considered “high activation” as activation of the output neuron over 50% and “low activation” as activation under 50%. The following interconnections between output pattern and the corresponding genotype were defined: high activation of output neuron *N*_1_ with simultaneous low activation of *N*_2_ represents the DDR2−/− genotype; high activation of *N*_2_ associated with low activation of *N*_1_ represents the DDR2+/+ genotype; while low activation of both *N*_1_ and *N*_2_ illustrates the heterozygous DDR2 genotype. To train neuronal network 1, datasets of skull images from 39 adult mice, 18 DDR2−/−, 8 DDR2+/− mice, and 13 wild-type control mice were included. The accuracy of the trained neuronal network 1 to predict DDR2 genotypes in mice was tested with an additional dataset of skull images of eight mice. As demonstrated in Table 2, all tested mice were successfully classified by this method. A 30% activation of neuron *N*_2_ indicates that a heterozygous DDR2 mouse displays only a mild skeletal malformation. All results were confirmed by standard genotyping techniques applying PCR with genomic DNA.

Network 1 was also shown to successfully discriminate between various two mice types. In particular this simple network could also distinguish between DDR1−/− mice and their wild-type controls as well as between SCID mice and C57BL/6, the wild-type littermates of DDR-deficient mice (unpublished data).

### ANN 2 Applied to fpVCT Datasets Discriminates DDR2−/−, DDR2+/−, DDR1−/−, C57BL/6 Wild-Type Controls, and SCID Mice between Different Mouse Populations

We applied our methodology to a larger cohort of mice derived from more than one strain of knockout mice. We used DDR1-deficient as well as SCID mice in combination with homogenous and heterozygous DDR2-knockout mice and their controls to train ANN 2. This multilayer perceptron network consists of one input layer with 34 neurons, two hidden layers with five and three neurons each, and one output layer with five neurons (Figure 4B). We included 49 mice in the training process including 18 DDR2−/−, six DDR2+/−, five DDR1−/−, and 16 C57BL/6 wild-type mice as well as four SCID mice. The experimental dataset consisted of skull images of 16 mice randomly chosen from the mouse cohort. As shown in Figure 4B, each mouse population was identified by one output neuron, *N*_1_ − *N*_5_. The output neuron, which shows maximal activation, determines the phenotype that the network estimates for a given input pattern independent of the values of the other output neurons. DDR2-deficient mice were classified with high accuracy as demonstrated by the most highly activated output neuron, *N*_2_, being consistent in all three cases (Table 3). Since skull features of DDR1−/−, DDR2+/−, and DDR1/2+/+ as well as SCID mice display only minor phenotypic differences among each other, these genotypes were more difficult to classify in a network with trained datasets from skulls of all five populations. This is shown by low activation of the corresponding output neurons, *N*_3_ for DDR1−/− and *N*_4_ for SCID mice, in comparison to the high activation observed in *N*_2_ for DDR2−/− mice. However, the transgenic status of DDR-deficient mice could be reliably predicted with the exception of DDR2 heterozygous mice and one SCID mouse, which were incorrectly classified as control mice by showing maximal activation of *N*_5_ (Table 3). It appears that the major differences in skull formation between DDR2−/− and control mice determine the network, thus making it more difficult to classify mice with phenotypes possessing only minor bone abnormalities.

**Table 3:**
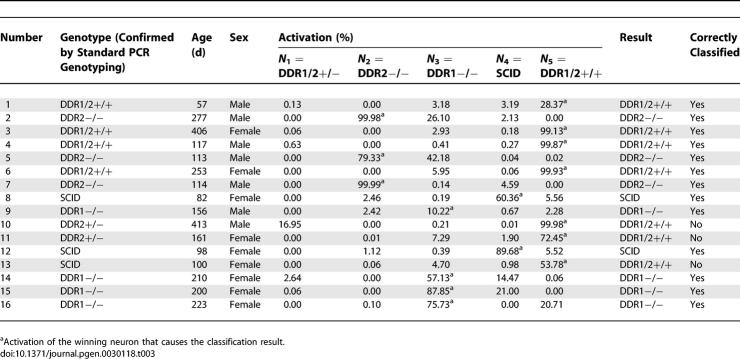
Discrimination of DDR1/2+/+, DDR2−/−, DDR2+/−, DDR1−/−, and SCID Mice between Different Mouse Populations Using fpVCT Datasets and ANN 2

### ANN 3 Applied to fpVCT Datasets Identifies DDR2+/−, C57BL/6 Wild-Type, and SCID Mice

ANN 3 was applied to distinguish between C57BL/6 wild-type control, DDR2+/−, and SCID mice that were identified in network 2 as control mice (Figure 4C). Neuronal network 3 received 34 input neurons and one output layer with two neurons, *N*_1_ and *N*_2_. This network also consisted of two hidden layer with five and three neurons. Low activation of both *N*_1_ and *N*_2_ represented SCID mice, high activation of output neuron *N*_1_ with simultaneous low activation of *N*_2_ represented C57BL/6 wild-type mice, whereas high activation of both *N*_1_ and *N*_2_ represented DDR2+/− mice. The network was trained with 21 datasets, seven DDR2+/− mice, nine C57BL/6 wild-type mice, and five SCID mice. As shown in Table 4, all mice were successfully classified by skull-based features using ANN 3.

**Table 4:**
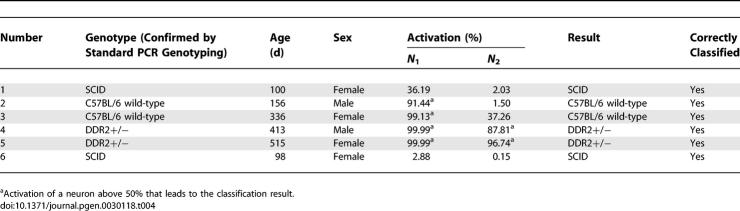
Identification of DDR2+/−, C57BL/6 Wild-Type, and SCID Mice Using fpVCT Datasets and ANN 3

### ANN 2 Applied to fpVCT Datasets Identifies DDR1/2 Double Knockout Mice

In an additional experiment we presented to the network 2 skull features of five DDR1/2 double knockout mice (DDR1−/−//DDR2−/−) without a new training process. Even when skull-shape features of these mice were not encoded in the network, the network response (Table 5) clearly depicted the existence of two skull shapes in all five mice tested, both related to DDR1- and DDR2-deficient mice. Therefore, the phenotype of the DDR double knockout mouse appears to be a superposition of the skull shapes from DDR1−/− and DDR2−/− mice.

**Table 5:**
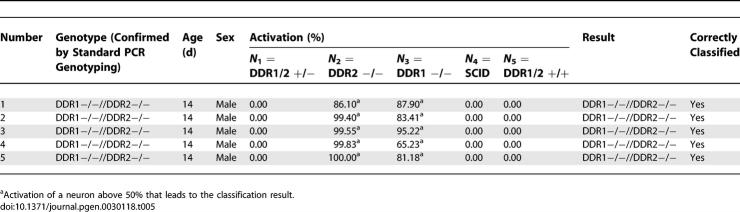
Identification of DDR1/2−/− Double Knockout Mice with ANN 2 That Is Only Trained for Single Knockout Mice

## Discussion

This study presents a rapid cost-effective primary screening method for comparing and identifying mutant mice with abnormalities in skeletal development out of the increasing number of mouse models that are now being generated where genes have been “knocked out,” “knocked in,” or mutated.

We have developed an ANN-based intelligent system for image interpretation of large 3-D fpVCT datasets. ANNs are interconnected groups of artificial neurons that use a mathematical or computational model for information processing based on a connectionist approach to computation [12]. High-resolution 3-D imaging fpVCT allows a detailed visualization of the mouse skeleton with clear contours. The delineation of the anatomical details of bone structures in mice by fpVCT imaging has been previously described [9].

Comparative morphological analysis is typically difficult in the skull and extremely laborious due to its very complex skeletal structure [6]. In our study, large 3-D fpVCT datasets were simplified to skull-shape features on the basis of high-order moments of the whole skull, making phenotyping of mouse models a much simpler, cost-efficient, and semi-automatic process [23]. One of the benefits of this semi-automatic classification method is that the manual work is reduced to the control of the segmentation process of skulls for which anatomical knowledge is not necessary, resulting in a rapid and therefore high-throughput application.

Since each of the 34 skull-shape features are based on all skull voxels, and therefore the features include all possible skull alterations, the method was found to be very reproducible and reliable for mouse classification. Therefore, in order to analyze novel phenotypes with skull abnormalities the same feature space can consistently be applied. Although, other statistical methods might be appropriate to analyze these complex biological relationships, we have chosen neuronal networks for the classification process because of several advantages of this method. Neuronal networks are able to elucidate nonlinear problems and learn by example, so the details of the complex morphological skull structure on the basis of which the mice classification is made, are not needed [12,13]. Therefore, the use of neuronal networks is highly favorable in our study, characterized by poorly conditioned features space and the comparison of mice types, in which the distinct alterations between the different skull phenotypes are not known yet.

Our results indicate that this computational-intelligence scheme based on 34 skull features is capable of identifying genetically modified mice with skeletal abnormalities observed on the five different trained mouse populations.

PCA and cluster analysis of these skull features were not able to discriminate between the different knockout mice demonstrating a poorly conditioned feature space and thereby showing that a nonlinear classification is a prerequisite to a correct determination of genetically altered mice models [15,16]. Here, features implemented in the ANNs were independent of skull sizes and orientations, thus making this method suitable to exclude interindividual variations of skull growth within mice of one group. Although features are related to age, this method is able to successfully discriminate between mice types when using mice older than 50 d. This goes in line with the observation that skull shapes of mice change with growth, but remain nearly constant 15 d postnatal [28]. The high significance of these features is also shown by the fact that even the smallest differences can be automatically detected, such as alterations in skull shapes related to sex. The algorithm was first tested on a cohort of DDR2-deficient mice with a known skeletal phenotype displaying shortage of long bones and a shorter snout [20]. In this study, this phenotyping method enables us to reliably detect DDR2-deficient mice within a cohort consisting of homozygous and heterozygous DDR2 mutants. Even heterozygous DDR2 mice with a subtle phenotype were correctly determined and identified as being different from their wild-type control by this method based on fpVCT imaging.

So far, no obvious skull abnormalities have been observed in DDR1-deficient mice. A reduced bone calcification has only been described in the fibula [21]. However, the imaging technique in combination with a neuronal artificial network trained only with skull-shape features generated from DDR1-deficient mice, and control mice were successful in discriminating clearly between DDR1-genotypes. With this method DDR1-deficient mice were identified as mice that show, in contrast to C57BL/6 wild-type animals, differences in skull formation. Landmark-based analysis of three distances and the occipital curvatures confirmed the presence of skull abnormalities of DDR2 knockout mice, thereby clearly defining a skeletal phenotype for these mutants. In contrast, DDR1-deficient mice with a subtle phenotype were not significantly altered in these features.

The screening tool based on skull-shape features is successful in discriminating between mouse strains displaying no overt differences in skull formation, for example distinguishing SCID mice from C57BL/6 wild-type mice. Even five double knockout mice for DDR1 and DDR2 not used in the training process, were identified by our semi-automatic classification method as a superposition of the classes related to the single features of DDR1−/− and DDR2−/− mice. This suggests that the algorithm discriminates not only between trained mice, but identifies different skull-shape traits.

The imaging technique, in combination with a more complex neuronal network, was also valuable to reliably discriminate DDR2-deficient mice between five different mouse populations, including SCID mice and DDR1-deficient mice. The challenge of creating a network for all presented mouse genotype-related phenotypes together is to balance the combined feature space of all classes. Therefore, the application of this ANN trained with features out of datasets from DDR2-deficient mice with a marked skeletal defect did not allow discrimination between the heterozygous DDR2-deficient mice, SCID mice, or their controls, which all show similarity in skull bones. However, thereafter they were successfully classified by applying the more specialized neuronal network for the three subtle mouse phenotypes.

In conclusion, we have introduced a novel semi-automatic screening method for skeletal phenotyping by applying neuronal networks in combination with fpVCT, so far limited to five different mice models. This methodology seems to be a powerful tool for the rapid detection of living mice with skull abnormalities. In the future, this technique is expected to be a standardized, cost-effective, primary screen to identify mice with skeletal differences out of a wide spectrum of genetically altered mice on the basis of random mutagenesis as well as transgenic and knockout mice. For successful identification of novel mutant mice with bone abnormalities, skull-shape features have to be calculated to create and train a novel neuronal network. However, because the introduced features are calculated automatically and include information of every skull voxel, they should easily be implemented to new skull shapes. Even a minor training error will indicate the existence of alteration in skull shapes. The degree of reliance to predict a skull phenotype is directly related to the correspondence of the neuronal network response for a second set of mice to their genotype. Therefore, skull alterations of genetic modified mice in comparison to their control littermates are depicted by correct classification of the mutant mice in a separate class.

Though successful in predicting the corresponding phenotype of various mouse populations in the primary screen, this methodology is not suitable for defining the exact bone deformation underlying the gene defect. The challenge of the next step will be to characterize the anatomical phenotypes in mice in more detail with stereological parameters by nondestructive visualization of complex skull structures by fpVCT imaging. Comparative analysis of multiple datasets of bone images will then allow to us to identify differences in corresponding anatomical sites between control and mutant groups, which has been demonstrated recently for MR imaging [6].

Since the overall shape of the skull depends upon coordinated development of separate bony, dental, and cartilaginous elements and functioning of soft tissue components, the identification of knockout mice with skull abnormalities and their characterization will help to further understand the role of major genes that are involved in the cascade of developmental processes necessary for the proper development of a functioning skull.

## Supporting Information

Protocol S1 C++ Implementation of Transformation and Feature Generation Part (6 KB TXT)

## Abbreviations

ANN: artificial neuronal network
CT: computed tomography
DDR: discoidin domain receptor
fpVCT: flat-panel volume computed tomography
PCA: principle component analysis
PCR: polymerase chain reaction
SCID: severe combined immunodeficient
